# Effect of a Mobile Phone Intervention on Quitting Smoking in a Young Adult Population of Smokers: Randomized Controlled Trial Study Protocol

**DOI:** 10.2196/resprot.3823

**Published:** 2015-01-19

**Authors:** Neill Bruce Baskerville, Laura Louise Struik, David Hammond, G Emmanuel Guindon, Cameron D Norman, Robyn Whittaker, Catherine M Burns, Kelly A Grindrod, K Stephen Brown

**Affiliations:** ^1^Propel Centre for Population Health ImpactLyle S. Hallman InstituteUniversity of WaterlooWaterloo, ONCanada; ^2^School of NursingUniversity of British Columbia's Okanagan CampusKelowna, BCCanada; ^3^School of Public Health and Health SystemsFaculty of Applied Health SciencesUniversity of WaterlooWaterloo, ONCanada; ^4^Centre for Health Economics and Policy AnalysisMcMaster UniversityHamilton, ONCanada; ^5^CENSE Research + DesignDalla Lana School of Public HealthUniversity of TorontoToronto, ONCanada; ^6^National Institute for Health InnovationUniversity of AucklandAucklandNew Zealand; ^7^Systems Design EngineeringUniversity of WaterlooWaterloo, ONCanada; ^8^School of PharmacyUniversity of WaterlooWaterloo, ONCanada; ^9^Statistics and Actuarial SciencesUniversity of WaterlooWaterloo, ONCanada

**Keywords:** health behavior, smoking cessation, young adult, mobile phone apps, mHealth

## Abstract

**Background:**

Tobacco use remains the number one cause of preventable chronic disease and death in developed countries worldwide. In North America, smoking rates are highest among young adults. Despite that the majority of young adult smokers indicate wanting to quit, smoking rates among this age demographic have yet to decline. Helping young adults quit smoking continues to be a public health priority. Digital mobile technology presents a promising medium for reaching this population with smoking cessation interventions, especially because young adults are the heaviest users of this technology.

**Objective:**

The primary aim of this trial is to determine the effectiveness of an evidence-informed mobile phone app for smoking cessation, Crush the Crave, on reducing smoking prevalence among young adult smokers.

**Methods:**

A parallel randomized controlled trial (RCT) with two arms will be conducted in Canada to evaluate Crush the Crave. In total, 1354 young adult smokers (19 to 29 years old) will be randomized to receive the evidence-informed mobile phone app, Crush the Crave, or an evidence-based self-help guide known as “On the Road to Quitting” (control) for a period of 6 months. The primary outcome measure is a 30-day point prevalence of abstinence at the 6-month follow-up. Secondary outcomes include a 7-day point prevalence of abstinence, number of quit attempts, reduction in consumption of cigarettes, self-efficacy, satisfaction, app utilization metrics, and use of smoking cessation services. A cost-effectiveness analysis is included.

**Results:**

This trial is currently open for recruitment. The anticipated completion date for the study is April 2016.

**Conclusions:**

This randomized controlled trial will provide the evidence to move forward on decision making regarding the inclusion of technology-based mobile phone interventions as part of existing smoking cessation efforts made by health care providers. Evidence from the trial will also inform the development of future apps, provide a deeper understanding of the factors that drive change in smoking behavior using an app, and improve the design of cessation apps. This trial is among the first to assess the effect of a comprehensive and evidence-informed mHealth smoking cessation app on a large sample of young adult smokers. Strengths of the trial include the high-quality research design and in-depth assessment of the implementation of the intervention. If effective, the trial has the potential to demonstrate that including mHealth technology as a population-based intervention strategy can cost-effectively reach a greater proportion of the population and help young adult smokers to quit.

**Trial Registration:**

ClinicalTrials.gov NCT01983150; http://clinicaltrials.gov/ct2/show/NCT01983150 (Archived by WebCite at http://www.webcitation.org/6VGyc0W0i).

## Introduction

### Background

Tobacco use remains the number one cause of preventable chronic disease and death in developed countries worldwide [1]. Currently, young adults represent the largest population of smokers across North America [2,3], and this age demographic is particularly vulnerable to the negative health effects of tobacco use [4-9]. Although smoking prevalence increases from adolescence into adulthood, most young adult smokers express a desire to quit. For example, Canadian young adults aged 20 to 24 and 25 to 34 who smoked reported that they were seriously considering quitting in the next 6 months at a prevalence of 61.7% and 71.5%, respectively [2]. Given evidence that quitting before the age of 40 reduces the risk of a tobacco-related death by as much as 90% [10], and that quit attempts decrease with age as patterns of tobacco use become engrained [2], helping young adults successfully quit smoking is a priority.

Finding effective solutions to help young adults quit smoking remains a challenge. Despite the existence of a myriad of evidence-based smoking cessation options [11], research suggests that younger adult smokers are particularly unlikely to seek treatment compared to older smokers [12-14]. For example, according to a survey investigating the use of cessation treatments, young adults aged 18 to 24 were half as likely to have used pharmacological (eg, nicotine replacement therapy NRT) or psychological (eg, advice from a health professional) treatments to aid with cessation as were older adults [14]. The underutilization of smoking cessation interventions by young adults combined with a lack of age-appropriate cessation interventions [15] and comprehensive marketing to younger populations by the tobacco industry [16] are major reasons for the lack of declines in young adult smoking rates. New strategies for reaching young adult smokers are needed. Recently, digital technologies have emerged as promising platforms to enhance tobacco control efforts directed toward this population [17].

Digital technologies have become ever more pervasive in young adults’ everyday lives. According to recent statistics, young adults aged 18 to 29 lead the way in the use of mobile phones, both those that run apps (65%) and those that do not (93%) [18]. The use of mobile phone apps has become a focused means for engaging young adults. Not only are young adults most likely to download apps, but they are also the most intense users of apps [19]. It is not surprising, then, that young adults are the most frequent users of health-related apps. It has been reported that 42% of those who seek health information through apps are young adults [20]. In addition, researchers have alluded to a trend toward increased use of mobile phones among lower socioeconomic status groups [21], increasing the likelihood of successfully delivering mobile phone-based health improvement interventions to traditionally hard-to-reach populations [22].

The use of mobile phone apps for health interventions, such as for smoking cessation, offers many unique benefits compared to traditional approaches, most notably because individuals can access these interventions anytime and in everyday settings [23] since assistance is immediately available when needed (eg, help in dealing with cravings). In addition, individuals have many opportunities to tap into various support networks [24] via their mobile phones, such as through social media. Support networks include those which are intervention related (eg, quit buddies and social networking sites associated with the intervention) and those related to their personal social networks (eg, personal contacts). In fact, social networks have been found to play a key role in young adults’ smoking cessation success [13,25,26]. Furthermore, the increasing use of internal sensors in mobile phones provides reliable contextual data that can infer such things as geographical location and has enabled tracking of health behaviors, as well as the delivery of interventions that are tailored to specific contexts [24]. These features enabled by mobile phones are a clear advancement over websites and short message service (SMS) text messaging programs. Their high potential to boost user engagement [27] has been consistently documented as a strong predictor of smoking cessation [28-30].

There is a growing body of evaluative evidence demonstrating that mobile phone-based technologies can support smoking cessation. However, most of this evidence consists of studies evaluating the efficacy of mobile phone SMS text messaging interventions for smoking cessation [31]. Young adults have reported an interest in more intense mobile-based smoking cessation interventions, such as mobile phone apps, versus what is currently offered via SMS text messaging, [12,32,33]. Mobile phone apps have the ability to enrich the user experience with more information, components, and functionality [34]. As well, smoking cessation mobile phone apps now have enormous reach compared to quitlines and SMS text messaging interventions [27]. For these reasons, exploring the effectiveness of mobile phone apps is critical. Only two randomized controlled trials (RCTs) were found that evaluated the efficacy of a smoking cessation mobile phone app. One compared a mobile phone app to an SMS text messaging intervention for smoking cessation [35]. It was reported in this study that both the mobile phone app and the SMS text messaging intervention predicted a significant increase in 30-day abstinence at 12 weeks [35]. Another pilot study tested the efficacy of a smoking cessation app based on acceptance and commitment therapy and found that it was feasible to deliver a theory-based mobile phone app with quit rates higher than the control condition [27]. Many mobile phone apps for smoking cessation exist that have the potential to integrate education, motivational techniques, quit plan assistance, linkages to support networks, quit coaches, and other functionalities that go beyond SMS text messaging [36]. Despite these functionalities, a recent systematic review reported that very few studies have been conducted to measure the outcomes of these apps, especially long-term outcomes [31]. A methodologically rigorous evaluation of mobile phone cessation technology is an identified gap in the published literature. The findings from this randomized controlled trial (NCT01983150) will help address this gap by determining whether mobile phone apps work for quitting and, more importantly, why they do or do not work. This is critical in light of an increasing push for mHealth scale-up [37].

### Study Aims

The primary aim of this study will be to determine the effectiveness of the evidence-informed mobile phone app for smoking cessation, Crush the Crave, on reducing smoking prevalence among young adult smokers after 6 months. Crush the Crave was developed by an expert team based at the University of Waterloo who worked with a technology development team to design, prototype, evaluate, and revise the program over the course of 2 years. This study will represent the first full trial of the program as part of a population-level intervention. We expect that individuals randomized to the evidence-informed mobile phone intervention condition will have higher 30-day point prevalence quit rates than individuals assigned to the control condition after 6 months.

Secondary aims of this study include the following:

1. Examine more proximal outcome measures of cessation behavior, including 7-day point prevalence abstinence (PPA), time to cessation, the number of quit attempts, and the reduction in consumption of cigarettes.

2. Examine satisfaction and patterns of app use at 3 and 6 months, including the extent to which it promotes the use of established smoking cessation services, such as nicotine replacement therapy, health professional consults, medications, and quitline counseling.

3. Examine mediators of cessation outcomes between conditions, such as frequency of app use, use of cessation services, quit intentions, nicotine withdrawal, and the following psychosocial mediators: beliefs and attitudes, stress, social norms, self-efficacy, and perceived social support.

4. Compare the cost-effectiveness of the intervention and control conditions.

## Methods

### Design

**Figure 1:**
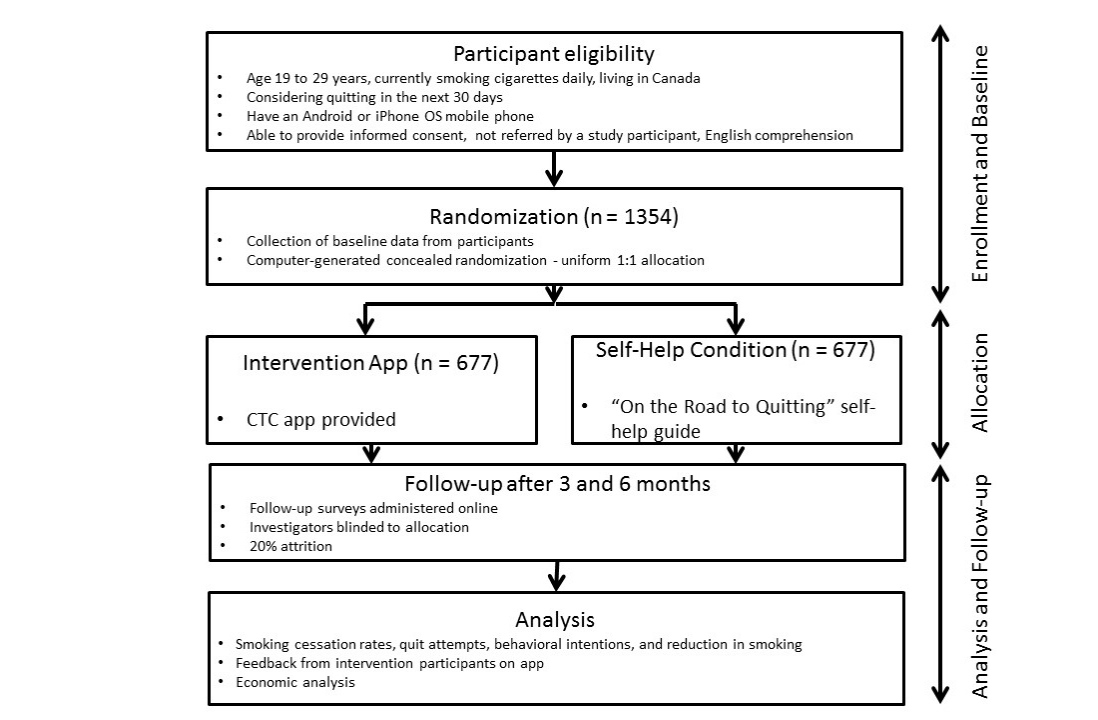
CONSORT-EHEALTH diagram of the study design.

This is a 6-month, two-arm, parallel randomized controlled trial (ClinicalTrials.gov Identifier: NCT01983150) to evaluate an mHealth intervention, Crush the Crave, for young adult smokers. Investigators and data collectors will be blinded to the group assignments. The protocol is in accord with the CONSORT-EHEALTH checklist [38]. See Figure 1 for a CONSORT-EHEALTH diagram of the proposed study design.

### Ethical Approval

The research methods to be used in this study have been approved by the Office of Research Ethics of the University of Waterloo (full ethics clearance granted on October 29, 2013, No. 19275).

### Study Population

Young adult male and female Canadian smokers are the target population for this study. Participants will be eligible for the study if they are between the ages of 19 and 29, are currently smoking cigarettes daily, are residing in Canada, are considering quitting smoking in the next 30 days, have an Android (version 2.0 to 5.0) or iPhone (version 4.0 to 7.0) OS mobile phone, are able to provide informed consent, are able to comprehend English, and have not been referred to the study by an existing study participant (eg, a friend or family member already participating in the study) to avoid possible contamination bias.

### Recruitment

Recruitment will be staggered over 32 weeks and will include online recruitment through Facebook advertisements and Kijiji, an online classified message board, with offline recruitment through classified newspaper ads, and a Can $35 incentive for registering for the trial. Interested young adults will be referred to a website describing the trial. Potential participants will be screened at the entry webpage where their eligibility will be determined, informed consent will be sought, and registration for the trial will take place. A total of 1354 young adult smokers will be recruited. After providing informed consent and having completed the online baseline questionnaire, a computer-generated email message will be sent back to the participants confirming registration and participants will then be randomly allocated to either the control or intervention arm. The control group will participate in a usual care, self-help guide intervention called “On the Road to Quitting” [39] and the intervention group will participate in the Crush the Crave program.

### Randomization and Blinding

Participants will be allocated to intervention and control groups using a uniform 1:1 (control:intervention) allocation ratio and a computer-generated, simple randomization procedure. We will monitor the comparability between groups and, given the large sample size, the groups will be balanced based on three sources of variability with regard to smoking—sex, age, and cigarette consumption [40]. As documented in similar large trials, the likelihood is remote that, by chance, the two study groups may not be well matched for baseline characteristics [41-43]. Owing to the nature of the intervention, participants will be aware of the group to which they have been assigned. However, investigators and data collectors will be blinded to group allocation until completion of the trial.

### Study Intervention

#### Overview

**Figure 2:**
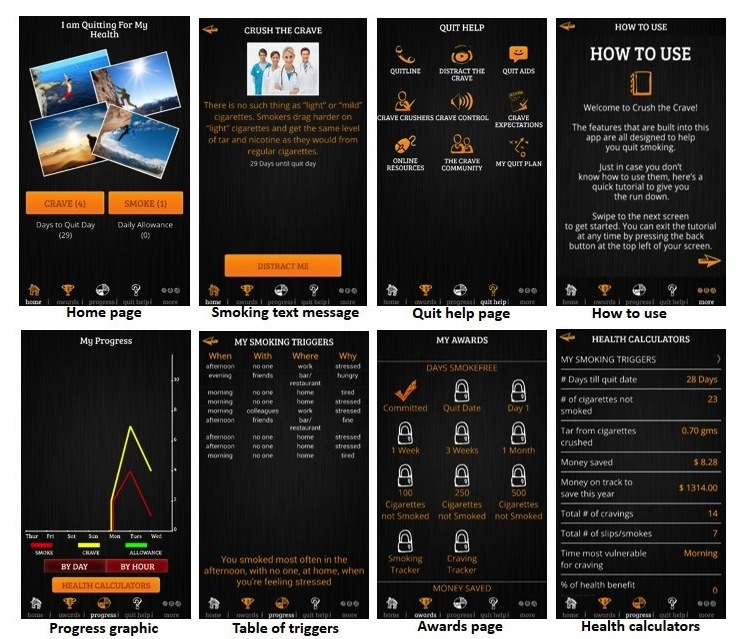
Screenshots of the quit-smoking mobile phone app, CrushtheCrave.

The intervention group will receive a comprehensive and evidence-informed smoking cessation mobile phone app, Crush the Crave, via the Internet. Crush the Crave enables users to customize a quit plan by choosing a quit date and then deciding whether to quit immediately or reduce the number of cigarettes they smoke every week up to their quit date. Crush the Crave then assists smokers in staying on track by reminding them of how much money they have saved and how much their health improves over time after quitting. Based on contingency reinforcement, these milestones are tracked as rewards, which smokers can then choose to share with their social network via Facebook and Twitter, and rally support from friends and family. Participants can also link to the Crush the Crave Facebook community for additional support for quitting. Users of the app also receive supportive text messages tailored to their specific quit plan and where they are in the quitting experience. Crush the Crave will allow the intervention group to track their daily smoking habits and cravings as well as understand their craving triggers or psychosocial determinants by recording when, where, and why they were smoking. The app provides both graphic and tabular performance feedback (see Figure 2). The app also provides online distractions to help smokers deal with their cravings. There are social media tools, such as a YouTube channel and opportunities to chat with friends online to distract a user until the craving subsides a few minutes later. Evidence-based information is available for assisting participants during the quitting experience on topics such as relapse and dealing with cravings. Furthermore, data are collected in real time to both support the user and to track the usage of the app allowing for push notifications, helpful reminders, and ongoing data collection. Finally, Crush the Crave provides access to evidence-based cessation services such as smoking cessation quitlines and explains the benefits of, and dispels myths around, nicotine replacement therapy.

#### Development of Crush the Crave

Crush the Crave was developed in early 2012 by a team of population health researchers, social media experts, and computer programmers as an evidence-informed, quit-smoking mobile phone and social media app for young adults aged 19 to 29. In addition to the input of key experts in the field of smoking cessation, development of the app incorporated Fiore’s practice guidelines for treating tobacco use and dependence [44] and principles of persuasive technology for behavior change [45]. The app was tested in March 2012 with eight focus groups consisting of 57 male and female young adult smokers on functionality, look and feel, and usability. Young adult smokers were engaged in the design and the naming of the app. Furthermore, the app was pilot-tested by over 300 smokers from April to June 2012. The pilot test revealed substantial engagement with 319 users triggering 7931 events, including 1444 visits to the quit help page, 1415 visits to the awards page, and 1016 visits to the progress tracking page. The focus groups of young adult smokers provided positive feedback on app functionality and content. In light of evidence that existing smoking cessation mobile phone apps are not developed by health professionals or academics, do not draw on behavior change theories or techniques, and do not have content aligned to clinical guidelines and other evidence-based practices [22,46], Crush the Crave is a relatively novel intervention in the area of smoking cessation.

### Standard Self-Help Condition

The control group will receive a standard, print-based self-help guide known as “On the Road to Quitting” [39] that has been recently developed by Health Canada for young adult smokers. This guide builds on an original guide that has been available for adult smokers for more than 10 years and includes evidence-based content for smoking cessation [47]. Participants will be able to both view and download the self-help guide via the Internet and can request a printed version of the guide. A print-based self-help guide was chosen as the control intervention because evidence from systematic reviews and meta-analyses of RCTs using printed self-help materials has demonstrated that there is no smoking cessation benefit from structured self-help printed materials [48-51].

Therefore, it has been determined that the effect of print-based self-help material is comparable to no treatment. In addition, a standard self-help control condition facilitates recruitment as it is problematic to offer participants a no-treatment option for a quit-smoking study.

### Data Collection

#### Baseline Questionnaire

Baseline data will be collected via a self-administered, online questionnaire completed by all consenting participants in both intervention and control groups. The baseline questionnaire will include the following demographic items: age, sex, ethnicity, marital status, education, income, and employment status. The following variables related to tobacco consumption will also be recorded: current smoking status, amount smoked, number and duration of past quit attempts, intentions to quit in the next 6 months, and the degree of nicotine dependence. Participants will also be asked a series of psychosocial questions, including beliefs and attitudes about quitting, self-efficacy or confidence in quitting, perceived stress and social support, and social norms related to smoking. Furthermore, participants will be asked about experience with mobile phone apps and self-help, use of NRT, and other cessation aids/supports, such as quitlines.

#### Questionnaire for 3- and 6-Month Follow-Up

Follow-up data will be collected from the same participants at 3 and 6 months in the same manner as the baseline questionnaire. In addition to the questions asked at baseline, participants will be asked core smoking status questions, including whether they have smoked any cigarettes or used other tobacco products, even a puff, in the last 30 days, 7-day point prevalence abstinence, number of quit attempts, and the reduction in cigarette consumption. Participants will then be asked questions on nicotine withdrawal, level of support received from friends and family for quitting smoking, additional cessation services that they sought for helping to quit, overall satisfaction with the app or self-help guide, use of the app or guide, opinions and beliefs about the app or guide, and challenges they experienced in quitting smoking. A modified Dillman method [52] for the online survey questionnaires will be used and up to 10 attempts (email and telephone) will be made to reach participants if they do not complete the online questionnaire within 2 weeks of the 3- and 6-month follow-up periods. Questionnaires will be pilot-tested with a convenience sample of young adult smokers.

### Outcome Measures

#### Primary Outcome Measure

The primary outcome measure will be self-reported, 30-day point prevalence abstinence from smoking at 6 months, operationalized as not having smoked any cigarettes, even a puff, or used other tobacco products in the last 30 days [53]. Biochemical validation of smoking status will not be done as a Cochrane Review of Internet-based interventions for smoking cessation found that very few studies used this method given the difficulties in obtaining samples [54]. In addition, accurate estimates of the prevalence of cigarette smoking among Canadians can be derived from self-reported smoking status data [55].

#### Secondary Outcome Measures

Secondary outcome measures are as follows:

1. The 7-day point prevalence abstinence at 6 months [53,56], the number of quit attempts (ie, how many times did participants stop using tobacco for 24 hours or longer over the past 6 months [57,58]), and the reduction in consumption of cigarettes [59].

2. Satisfaction, app utilization metrics, such as frequency of use and use of smoking cessation services (eg, NRT, health professional consults, medications, quitline counseling, and e-cigarettes).

3. Beliefs and attitudes, stress [59], social norms [57], behavioral intentions to quit smoking [58], degree of nicotine dependence and nicotine withdrawal [60], self-efficacy [61,62], and perceived social support [61,62].

Recent research on e-cigarette use has found the use of e-cigarettes is increasing rapidly, and research has found evidence of dual-use. Young adult smokers do not necessarily view e-cigarettes as cessation aids, as some consider them a complement and substitute for smoked cigarettes [63,64]. The prevalence of e-cigarette use amongst the cohort of young adult smokers, and the extent to which e-cigarette use mediates the primary outcome, will be investigated.

### Process Measures

For process measures, we will monitor downloading of the app and app usage via the tracking of events triggered when participants use the app (eg, connects with social support, seeks information on smoking, and tracks progress). For tracking, we will use Web-analytic data via Google Analytics and a secure database of app usage which records events triggered with a time stamp. For example, on a per-user basis we will track when a user logs into the app, number of user clicks on the “Smoke” or “Crave” buttons, and achievement of awards. Aggregate Google Analytics metrics will include visits, page view counts, and average time spent on a page.

To obtain a more in-depth understanding of the potential barriers to, and facilitating factors of, uptake of the mobile phone intervention, we will conduct semistructured interviews with a subsample of participants in the intervention group. To maximize variation (eg, age, sex, and level of satisfaction with the app), participants will be purposively recruited via email or telephone to participate. Sampling will be driven by saturation of themes. In keeping with previous studies [65], we anticipate the need to conduct approximately 40 interviews. Interviews will take place via telephone by a project team member with experience in qualitative interviews and will last 30 to 45 minutes. We will seek feedback on the app utilization, opinions and beliefs about the app, acceptability of the mobile phone intervention, participants’ specific likes and dislikes, and their perceived utility and satisfaction with each of the mobile phone intervention components. Interviews will be digitally recorded and transcribed. Interview transcripts and sociodemographic data will be entered as a project in the qualitative data software program, NVivo version 10. Study team members trained in qualitative methods will use an iterative process to understand the themes and key issues arising from the data.

### Sample Size and Power Calculations

Sample size calculations are based on a superiority trial [66,67] and are focused on the difference in the objective measure of the primary outcome event—30-day point prevalence abstinence from smoking—between intervention and control groups. Assuming a ratio of 1:1 for intervention to control subjects, an alpha of .05, power of 80%, and an effect size equal to a 50% increase in self-reported abstinence—17% in the intervention versus 11% in the control condition—the required sample size is 524 per group, for a total of 1048 participants using a two-tailed test on proportions [68]. The 50% increase in effect is reasonable and conservative based on trials of interventions using other mobile and Web-based technologies [41,69,70]. The estimated 11% abstinence rate in the control condition is based on the Ontario Tobacco Survey young adult smokers cohort study where in a sample of 592 young adult smokers, 68 (11.5%) were abstinent at the 6-month follow-up [71]. The 11% abstinence rate is what is expected to occur with a standard self-help condition and is similar to other trials that have tested the effect of self-help materials, with validated 6-month cessation rates of 5% to 15% [50,51]. Based on the experience of similar interventions [41,69], an overall estimate of 20% attrition translates into quit rates of 8.8% in the control condition and 13.6% in the intervention. Therefore, the total sample size required for each group is 677, for a total of 1354 participants.

### Analysis Plan

#### Statistical Analysis

The intention-to-treat principle will be followed for statistical analyses and the imputation method for missing data will be conservative whereby nonresponders will be classified as smokers. Characteristics will be compared between the groups at baseline, at 3-month follow-up, and at 6-month follow-up using independent *t* tests for continuous variables, or chi-square tests for categorical variables as appropriate. Mann-Whitney U tests will be used where data are not normally distributed. Preliminary analyses using models appropriate for the distribution of the variables will be undertaken to determine variables that differ between treatment groups, and between those with complete and incomplete follow-up data. Any variables showing a significant difference will be used in models as covariates to adjust for imbalances between the groups.

The primary response variable—30-day abstinence at 6-month follow-up—will be analyzed in a logistic regression model with the treatment group as the main explanatory variable. Age, sex, and level of addiction will be included, as well as other covariates, to adjust for any differences between the groups. The coefficient for the treatment group will be tested at the 5% level using a likelihood ratio test. Evidence against the null hypothesis that the coefficient is zero will indicate a significant difference between the treatment groups, after adjustment for covariates that differ between the groups.

Secondary analyses that involve comparisons of 7-day abstinence, quit attempts, and consumption levels will be conducted using generalized linear models that consider the distribution of the response variable (eg, Poisson models for numbers of quit attempts) and special features of the data (eg, zero-inflated Poisson models for quit attempts, if necessary). Similarly, variables such as satisfaction, use of e-cigarettes, and use of cessation services will be modeled using generalized linear regression models that include the indicator variable for condition.

**Figure 3:**
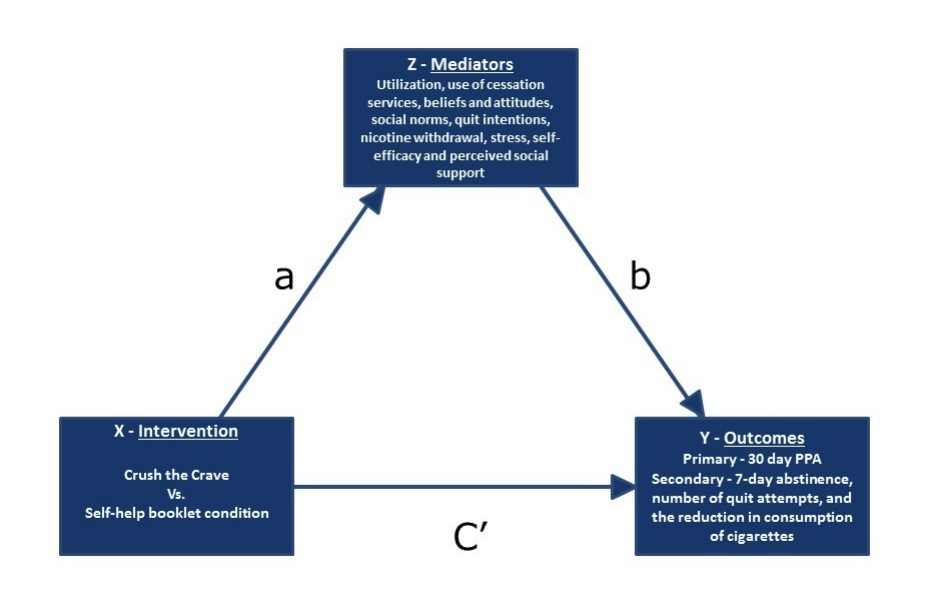
Mediation model. Conceptual diagram depicting Intervention (X), Outcome (Y), and Mediator (Z) variables, as well as hypothesized relationship (a), action (b), and outcome (C') pathways for examination in the mediation analyses.

The Preacher and Hayes bootstrapping method will be used to provide point estimates and confidence intervals to assess the significance or nonsignificance of a mediation effect, as it provides an increase in power and does not require the normality assumption to be met [72]. The mediation analyses will proceed by first estimating the difference in the outcome variable between the treatment and control groups, adjusting for age and sex as necessary, and then estimating the difference between the conditions for the mediating variables using bootstrapping. Finally, mediating variables and the treatment group indicator, along with covariates, will be entered into models for the primary and secondary responses. This will allow for an investigation of the role the mediators play in accounting for the differences between conditions. For example, use of e-cigarettes, higher levels of dependence, lower levels of self-efficacy, and low perceived social support might mediate quit success, for example, lower quit rates (see Figure 3). Additional secondary analyses will look at models for the response variables involving a treatment group indicator, covariates, and interactions between covariates and the treatment group indicator. These models will examine whether the relationships between the outcome and covariates differ between the conditions. They will also help to identify potential mechanisms, for example, frequency of app usage, by which the intervention might be operating. Statistical analyses will be performed using SAS version 9.4.

#### Economic Analysis

The lifetime incremental costs and benefits of the Crush the Crave mobile phone app added to current practice will be estimated from a government perspective using a Markov model adopted in previous economic evaluations of smoking cessation interventions [73]. All costs associated with development of the app and research will be excluded. The costs of delivering the intervention will be assessed by measuring and valuing the incremental resources used [73], including the costs of app maintenance (eg, costs to maintain the server and keep the app working on new operating systems) and the cost of the moderator for the social networking component. A number of brief questions will be included in the follow-up questionnaire concerning the use of NRT, quitlines, and other cessation services to ascertain these costs. Following a methodology proposed by Drummond et al [74], cost-effectiveness will be measured in terms of cost per quitter (6-month continuous abstinence—30-day point prevalence), cost per life year gained, and cost per quality-adjusted life year (QALY) gained for smoking-related diseases—lung cancer, stroke, myocardial infarction, chronic obstructive pulmonary disease, and coronary heart disease—between the intervention and control groups. The incremental cost-effectiveness ratio will also be measured in a manner similar to other studies [75]. To account for the timing of events, costs and consequences will be discounted at 3% [74]. To ensure the robustness of our cost-effectiveness estimates, we will conduct multivariate sensitivity analysis using Monte Carlo simulation. Key input parameters to be examined will include, but will not be limited to, discount rate, intervention costs, quit rates, and unit costs of smoking-related diseases.

## Results

This trial is currently open for recruitment. The anticipated completion date for the study is April 2016.

## Discussion

### Principal Findings

The Crush the Crave trial will evaluate a comprehensive evidence-informed mobile phone intervention for reducing smoking prevalence among a large sample of young adult smokers. To the best of our knowledge, this is one of three published mHealth protocols to assess the effect of a comprehensive and evidence-informed mobile phone app for smoking cessation, and one of the first to evaluate the impact of mHealth smoking cessation self-management on a large sample of young adult smokers. At the time of writing, there were eight trials underway regarding smoking cessation and mobile phone apps (ClinicalTrials.gov) and only two studies that have published findings on the effect of mobile phone-delivered smoking cessation interventions [27,35]. Bricker et al [27] conducted a double-blind randomized trial with a small sample of adult smokers on a theory-based mobile phone app as the intervention versus the National Cancer Institute’s smoking cessation app as the control and found overall quit rates of 13% in the intervention condition versus 8% in the control condition. Conversely, Buller et al [35] compared a mobile phone app with SMS text messaging for a small sample of young adult smokers. They found that the mobile phone app intervention was feasible for delivering cessation support but did not appear to move smokers to quit as quickly as SMS text messaging. The large sample of young adult smokers participating in the Crush the Crave trial will allow for a determination of what factors mediate quitting success through the use of an evidence-informed mobile phone app. In addition, it is one of very few studies to our knowledge that considers the cost-effectiveness of an mHealth intervention for smoking cessation among young adults [75].

Common criticisms have been made regarding mHealth research designs and trial descriptions [76]. Few large samples and adequately powered randomized controlled trials have been completed to date and many of those that have been done are of short duration and do not fully describe or assess the implementation of the mobile phone intervention. The Crush the Crave trial has been developed to address these concerns with a rigorous design, large sample size, 3- and 6-month follow-up periods, and attention to monitoring the process of implementation and utilization of the intervention. This will allow for sufficient explanation so that others can replicate the intervention [38]. As mHealth technology evolves rapidly, the Crush the Crave trial will allow researchers and policy makers to know which aspects of the intervention worked and which did not [77]. In addition, the development of Crush the Crave involved the target audience and it has been suggested that engaging the users of mHealth interventions is a contributing factor to their adoption and success [78].

### Limitations

A limitation of this study is the lack of an effect size for the control group self-help condition for determining sample size. However, systematic reviews and RCTs of printed self-help interventions for young adult populations support the effect size estimate chosen [48-51].

### Conclusions

It is evident that the young adult population of smokers is interested in mHealth technology for helping them quit smoking [24]. If the proposed trial finds support for effective delivery of smoking cessation interventions via mobile phone apps to help young adults quit, it would provide evidence to move forward and include technology-based interventions as part of existing smoking cessation efforts made by health care providers. It would also inform the development of future apps, provide a deeper understanding of the factors that drive change in smoking behavior, and improve the design of existing apps. This study will provide data on the potential of including mHealth technology in population-based smoking cessation interventions as a strategy to economically and effectively reach young adults, and ultimately reduce the prevalence of smoking in this age demographic.

## Abbreviations

NRT: nicotine replacement therapy
PPA: point prevalence abstinence
QALY: quality-adjusted life year
RCT: randomized controlled trials
SMS: short message service
